# Association between exposure to a mixture of organochlorine pesticides and hyperuricemia in U.S. adults: A comparison of four statistical models

**DOI:** 10.1016/j.eehl.2024.02.005

**Published:** 2024-03-05

**Authors:** Yu Wen, Yibaina Wang, Renjie Chen, Yi Guo, Jialu Pu, Jianwen Li, Huixun Jia, Zhenyu Wu

**Affiliations:** a School of Public Health, Key Laboratory of Public Health Safety and Collaborative Innovation Center of Social Risks Governance in Health, Fudan University, Shanghai 200032, China; b China National Center for Food Safety Risk Assessment, Beijing 100022, China; c National Clinical Research Center for Ophthalmic Diseases, Department of Ophthalmology, Shanghai General Hospital, Shanghai Jiao Tong University, School of Medicine, Shanghai Key Laboratory of Fundus Diseases, Shanghai 200080, China

**Keywords:** Hyperuricemia, Organochlorine pesticide, NHANES, Weighted quantile sum, Quantile g-computation, Bayesian kernel machine regression

## Abstract

The association between the exposure of organochlorine pesticides (OCPs) and serum uric acid (UA) levels remained uncertain. In this study, to investigate the combined effects of OCP mixtures on hyperuricemia, we analyzed serum OCPs and UA levels in adults from the National Health and Nutrition Examination Survey (2005–2016). Four statistical models including weighted logistic regression, weighted quantile sum (WQS), quantile g-computation (QGC), and bayesian kernel machine regression (BKMR) were used to assess the relationship between mixed chemical exposures and hyperuricemia. Subgroup analyses were conducted to explore potential modifiers. Among 6,529 participants, the prevalence of hyperuricemia was 21.15%. Logistic regression revealed a significant association between both hexachlorobenzene (HCB) and trans-nonachlor and hyperuricemia in the fifth quintile (OR: 1.54, 95% CI: 1.08–2.19; OR: 1.58, 95% CI: 1.05–2.39, respectively), utilizing the first quintile as a reference. WQS and QGC analyses showed significant overall effects of OCPs on hyperuricemia, with an OR of 1.25 (95% CI: 1.09–1.44) and 1.20 (95% CI: 1.06–1.37), respectively. BKMR indicated a positive trend between mixed OCPs and hyperuricemia, with HCB having the largest weight in all three mixture analyses. Subgroup analyses revealed that females, individuals aged 50 years and above, and those with a low income were more vulnerable to mixed OCP exposure. These results highlight the urgent need to protect vulnerable populations from OCPs and to properly evaluate the health effects of multiple exposures on hyperuricemia using mutual validation approaches.

## Graphical abstract

**Figure:**
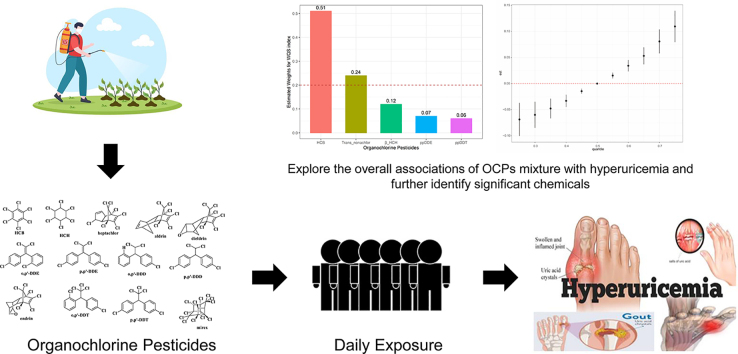

## 1 Introduction

Uric acid (UA) is the end product of purine metabolism in the human liver [1,2]. The concentration of UA in the blood is determined by the equilibrium between its production (from dietary purine ingestion or endogenous synthesis) and elimination (renal [65%–75%] or gastrointestinal [25%–35%] excretion) [3]. Hyperuricemia may develop when the serum UA level exceeds the normal range, posing a significant public health concern due to its high prevalence and heavy disease burden. Across diverse populations, the reported prevalence of hyperuricemia varies from 8.9% to 24.4% [4], and the serum UA concentration is substantially associated with cardiovascular and all-cause mortalities. This includes a 39% rise in total cardiovascular mortality and a notable 35% rise in ischemic stroke mortality [5].

Various risk factors contribute to hyperuricemia, including genetics, obesity, consumption of alcohol, and a purine-rich diet, as established by previous epidemiological studies [[6], [7], [8], [9]]. However, these factors do not fully account for the development of hyperuricemia [10]. Recently, attention has shifted to the impact of environmental contaminants, such as air pollutants, heavy metal pollutants, and persistent organic pollutants (POPs), on hyperuricemia [1,[11], [12], [13]]. Organochlorine pesticides (OCPs), considered typical POPs, were historically commonly used to control agricultural pests due to their inexpensive cost and high effectiveness. However, OCPs show a notable resistance to degradation and a tendency to accumulate and undergo biomagnification throughout the food chain [14]. Exposure to these pollutants might damage glomerular and tubular renal cells. Siddharth et al. reported an inverse correlation between blood OCP concentration and estimated glomerular filtration rate (eGFR). Such a correlation might diminish the capacity to eliminate UA [15], ultimately leading to hyperuricemia and gout. Moreover, several previous studies have suggested a potential link between OCPs and hyperuricemia in the general population. For example, cross-sectional studies from both the U.S. and Korea found elevated serum OCP levels increased the risk of hyperuricemia [16,17]. However, these population-based studies are limited by either focusing on a single OCP or having a relatively small sample size, potentially biasing the conclusions.

A notable concern in previous studies is the methods used to analyze the health effects of pollutants. Typically, logistic regression is employed to evaluate the impact of individual OCP exposure on hyperuricemia [1,12,16,17]. The limitation of this conventional analytical framework lies in its disregard for the combined effect and the high correlations among chemicals, presenting challenges for adopting more sophisticated analytical methodologies [10,11,18]. Weighted quantile sum (WQS) regression is proposed as an effective method for evaluating the combined effects of chemical mixtures. However, the utilization of WQS is also limited by the linear and directional homogeneity assumption of the model [19]. In recent years, quantile g-computation (QGC) has been employed to overcome the limitations posed by the unidirectional assumption of WQS regression [20]. In this study, we also employed a novel semi-parametric modeling method, bayesian kernel machine regression (BKMR), to capture the combined effects of mixture components which allows for possible interactions and nonlinear effects. Recent evidence also supports the utilization of BKMR to estimate the cumulative health effects of chemical mixtures because BKMR can investigate 1) the combined effect of chemical mixtures, 2) the separate concentration–response relationships of each mixture component adjusted for the others, and 3) the relative contributions of various pollutants to the overall impact [[21], [22], [23]]. However, to our knowledge, BKMR has yet to be explored in the context of OCP mixtures and the onset of hyperuricemia.

Our objective was to identify the associations of multiple OCPs with hyperuricemia, leveraging data from the National Health and Nutrition Examination Survey (NHANES). We analyzed the effects of the chemicals using logistic, WQS, QGC, and BKMR models. Each of the four models presents its own strengths and weaknesses, and we have integrated these results for a comprehensive interpretation.

## 2 Materials and methods

### 2.1 Study population

NHANES, administered by the National Center for Health Statistics (NCHS) of the CDC, aims to obtain nationally representative information on the civilian, noninstitutionalized population of the U.S. Participants in the survey underwent a series of exhaustive evaluations, which encompassed in-depth home interviews, thorough physical examinations, rigorous laboratory testing, and detailed medical history reports. The data used in our study were sourced from the NHANES. As part of its standard protocol, NHANES undergoes a thorough ethical review and obtains approval from the NCHS Ethics Review Board (ERB) for each survey cycle. This includes comprehensive evaluations of the survey methodologies, data collection procedures, and the ethical implications associated with the use of human participants in research. In the case of individuals under the age of 18, consent was obtained from their legal guardians [24].

This study incorporated data from the six most recent consecutive cycles of the NHANES from 2005–2006 to 2015–2016 (the datasets were publicly available from the CDC website) [25]. We focused on 6,529 qualified participants who were over 20 years of age, in line with previous comparable studies [[26], [27], [28], [29]]. Other inclusion criteria include complete measurements for serum UA and OCPs, and complete crucial covariates excluding pregnancy (Fig. S1).

### 2.2 Exposure assessment and target OCPs

Prior to analysis at the National Center for Environmental Health, serum samples were stored at low temperatures. The samples were processed using an automatic liquid handler (Gilson 215 Liquid Handler®, Gilson, Inc., Middleton, WI, U.S.). Following this, they were extracted through liquid/liquid extraction and then eluted from a column containing 0.25 g silica and 1 g silica/sulfuric acid (33% by weight) using a solution of 5% DCM in hexane (10 mL). The target analytes were identified using isotope dilution gas chromatography–high resolution mass spectrometry (GC/IDHRMS). Lipid weight (ng/g lipid) was used to present the concentrations of the target analytes. More information about the laboratory methods can be found on this website: https://wwwn.cdc.gov/nchs/nhanes/ (accessed on September 20th, 2023).

Because serum levels of OCPs were measured only between 2005 and 2016, our study included data from survey cycles within this timeframe [30]. β-hexachlorocyclohexane (β-HCH), hexachlorobenzene (HCB), p,p′-DDE, p,p′-DDT, trans-nonachlor, Mirex, and Oxychlordane were measured during each of the six survey cycles, while γ-hexachlorocyclohexane (γ-HCH) was measured from 2005 to 2012 and o,p′-DDT was measured from 2005 to 2008. In cases where OCP concentrations were below the detection limit, a value equal to the detection limit divided by the square root of 2 was assigned. In summary, based on our previous research, we selected OCPs with a detection frequency greater than 60% as the target OCPs (including β-HCH, HCB, p,p′-DDE, p,p′-DDT, and trans-nonachlor) for the final analysis and presented results based on lipid-adjusted concentrations (ng/g lipid) [31].

### 2.3 Hyperuricemia assessment

Serum UA concentrations, a typical component of the standard biochemistry profile, were measured with the Cel DxC800 Synchron. Diagnostic criteria for hyperuricemia were established with thresholds of 7 mg/dL for males and 6 mg/dL for females [[32], [33], [34]].

### 2.4 Covariates

Selected covariates were considered based on prior studies [10,11,27], including age, sex, race (Hispanic-Americans, non-Hispanic Whites, non-Hispanic Blacks, and others), education level (below high school, high school, and above high school), body mass index (BMI), serum cotinine concentration, alcohol consumption, serum creatinine concentration, poverty income ratio (PIR, < 1.3, 1.3–3.5, and > 3.5) [27], physical activity (< 600, 600–1,199, and ≥ 1,200 MET min/week), total calories intake (low: males < 2,000 kcal/day, females < 1,600 kcal/day; adequate: males 2,000–3,000 kcal/day, females 1,600–2,400 kcal/day; and high: males > 3,000 kcal/day, females > 2,400 kcal/day), medical history (diabetes, hypertension, and chronic kidney disease [CKD]), serum UA concentration, eGFR and total frequency of seafood consumption. Age, serum cotinine concentration, serum creatinine concentration, serum UA concentration, and eGFR are continuous variables. The total frequency of seafood consumption is defined as all the frequencies of fish, shellfish, crabs, crayfish, and lobsters consumed during the past 30 days, and was converted into quintiles [35,36]. Alcohol consumption is defined as consuming at least 12 alcoholic drinks per year or not, and eGFR is calculated based on the CKD-EPI (Chronic Kidney Disease Epidemiology Collaboration) equation [37].

### 2.5 Statistical analyses

Since the levels of all OCPs were right-skewed, natural logarithms (ln) were calculated when these variables were treated as continuous variables and transformed to improve statistical models for data normality. The *t*-test and *χ*^2^ test were utilized to compare continuous and categorical variables, respectively. The Spearman correlation coefficients *r*_*s*_ between natural logarithm-transformed concentrations of the five chemicals were also computed.

To analyze the individual and combined effects of OCPs, we employed weighted multiple logistic regression, WQS regression, and QGC model. Subsequently, we utilized BKMR to validate both the univariate response relationships and the combined impact. Lastly, sub-group analyses were conducted to further explore potential effect modifiers, including sex, age, and PIR.

#### 2.5.1 Weighted multiple logistic regression

OCP concentrations were included both in the continuous and quintile forms in the regression models to reduce the residual confounding [38], with the lowest quintile serving as the reference category. The investigation utilized odds ratios (ORs) and 95% confidence intervals (CIs) to evaluate the magnitude of the associations between each OCP variable and hyperuricemia. Linear trends between each OCP and the risk of hyperuricemia were evaluated by utilizing median concentrations in each quintile group. Sample and subsample weights were designed to adjust for the complex survey design to ensure the estimations were representative of the U.S. general population [30]. Thus, survey-weighted logistic models, specifically designed for NHANES, were used in the multiple regression analyses. In addition, three logistic models were constructed for each OCP: The initial model, referred to as Model 1, lacked any confounding factors. By contrast, Model 2 was enhanced by incorporating adjustments for age, sex, race, education level, PIR, and BMI. Lastly, Model 3 was built upon Model 2 by additionally accounting for alcohol consumption, serum cotinine concentration, physical activity, total calorie intake, and medical history (diabetes, hypertension, and CKD). To address the potential increased type I error caused by multiple testing, we applied false discovery rate (FDR) corrections in the context of multiple testing [39].

#### 2.5.2 Weighted quantile sum regression

We used WQS regression to align the shared directionality of all target OCPs towards hyperuricemia. This allowed us to assess the joint impact of five OCPs on hyperuricemia and the individual contributions of each pollutant. The WQS regression model classifies OCPs as quartile-based ordered variables and computes the WQS index that represents the total burden of the five OCPs. The contributions of each OCP to the WQS index are indicated by their respective weights. Afterward, the association between overall combined OCP exposure and the outcome is simulated using a linear modeling framework [40,41], as below:

$$g\left(\mu \right)=\beta_{0}+\beta_{1}\left(\sum_{i=0}^{c}\omega_{i}q_{i}\right)+Ζ\phi$$

$$\text{WQS}=\sum_{i=0}^{c}\omega_{i}q_{i}$$

where *β*_0_ is the intercept; *ω* represents the anticipated weight estimation of the *i*-th OCP to be determined; *q_i_* represents the different quartiles of OCP score values (*q_i_* = 0, 1, 2, or 3 corresponds respectively to the first, second, third, and fourth quartiles); *c* represents the number of OCPs included in the analysis (here, five); *β*₁ is the regression coefficient; *Ζ* is the covariate vector; *ϕ* is the covariate regression coefficient vector; and *g(μ)* is the logit link function for hyperuricemia *vs*. non-hyperuricemia.

In the current study, the data were randomly divided into two subsets: a training set consisting of 40% of the data and a validation set consisting of the remaining 60%. The value of *β*_1_ was specified as positive [40]. Additionally, the training set underwent 1,000 bootstrap resampling iterations, obtaining 1,000 sets of *ω*_*i*_ and *β*_1_. When *β1* was positive and convergence was attained within-group iterations, empirical weights were computed by averaging these weights. These empirical weights were then used to determine the significance of the validation set.

Our study involved fitting three WQS models. Model 1 was a simplistic model without covariates. Model 2 was modified for age, sex, education level, PIR, race, and BMI. Further adjustments were made to Model 3, including serum cotinine concentration, alcohol consumption, physical activity, total frequency of seafood consumption, total calorie intake, and medical history (diabetes, hypertension, and CKD). WQS is implemented using “gWQS” in R.

#### 2.5.3 Quantile g-computation

We utilized QGC [20] to evaluate the association between a mixture of OCPs and hyperuricemia, as well as to determine the individual contributions of various OCPs to hyperuricemia. In QGC, OCPs were converted to quartiles, and the following linear model was fitted (covariates omitted):

$$Y_{\text{hyperuricemia}}=\beta_{0}+\sum_{j=1}^{p}\;\beta_{j}{\text{OCP}}_{j}^{q}+\varepsilon_{i}$$

where *β*_0_ indicates the model intercept, ${OCP}_{j}^{q}$ is the quartile form of *OCP*_*j*_, the error term is *ε*_*i*_, and the weighted quantile sum is $\sum_{j=1}^{p}\;\beta_{j}$, which shows the change in hyperuricemia level per unit change in all OCPs. The weight is computed for each OCP, denoting whether it contributes positively or negatively to hyperuricemia. The weight is computed for each OCP, indicating whether it contributes positively or negatively to hyperuricemia. Specifically, for positive OCPs, the weight is equal to 1; for negative OCPs, the weight is equal to −1.

QGC demonstrated the ability to assess the combined effect of chemicals with multiple variables in various directions by integrating the flexibility of g-computation with the simplicity of inference offered by the WQS regression model. In our study, we employed the QGC model to estimate the change in the prevalence of hyperuricemia for a simultaneous one-quantile increase in the five OCPs [42,43]. We performed 200 bootstraps and utilized involved three QGC models. Model 1 was a simplistic model without covariates. Model 2 was modified for age, sex, education level, PIR, race, and BMI. Further adjustments were made to Model 3, including serum cotinine concentration, alcohol consumption, physical activity, total frequency of seafood consumption, total calorie intake, and medical history (diabetes, hypertension, and CKD). QGC is implemented using “qgcomp” in R.

#### 2.5.4 Bayesian kernel machine regression

BKMR is a non-parametric Bayesian variable selection method. Using iterative regression of high-dimensional exposure-response functions with a Gaussian kernel, we evaluated the overall relationship between chemical mixtures and outcomes [21,22], leveraging the following equation:

$$Y_{i}=h\left(z_{i}\right)+x_{i}^{T}\beta +\varepsilon_{i}$$

where *Y*_*i*_ represents the health endpoint; *h*(.) signifies a high-dimensional exposure-response function, permitting non-linearities and interactions between combined chemicals; *x*_*i*_ is potential confounders; *z*_*i*_ is the chemical exposures; *β* indicates the confounding coefficients; *ε*_*i*_ is the residual; and *i* is the individual (*i* = 1, 2, 3 … *n*).

For combined exposures and binary results (hyperuricemia or not), we postulated a probit link function. In the context of BKMR, employing probit regression requires the representation of the exposure-response association between the exposure variable and the latent continuous outcome as denoted by the function *h*(.).

The model was fitted utilizing a Markov chain Monte Carlo (MCMC) approach, with a total of 20,000 iterations [11,22,44], to ensure the robustness and accuracy of our findings. The BKMR model demonstrates efficacy in considering intricate nonlinear and additive associations between OCP combinations and hyperuricemia. The BKMR model utilizes kernel functions to investigate the cumulative correlation between the mixture and its results, as well as the high-order interaction between a single component and numerous components of the mixture. In this study, we used BKMR to visualize: (1) univariate response relationships for single OCPs and hyperuricemia while keeping four other OCPs fixed at their respective median concentrations; and (2) the impact of OCP mixtures on hyperuricemia across different quantiles, in comparison to administering all mixtures at their median concentrations. BKMR is implemented using “bkmr” in R.

In addition to the BKMR approach, we employed the Cross-validated Ensemble of Kernels (CVEK) method to further assess the interaction effects among organochlorine pesticides (OCPs). CVEK, by integrating a cross-validated ensemble of kernels within the kernel machine regression framework, allows for a more nuanced estimation of the complex nonlinear interactions [23,45]. CVEK was implemented using “CVEK” in R.

#### 2.5.5 Sub-group analyses

We further conducted stratified analyses to examine the association between OCP exposure and hyperuricemia in various subgroups, including sex (male or female), age (< 50 or ≥ 50 years), and PIR (< 1.3, 1.3–3.5, and > 3.5) [26,28,29,46]. The multiple logistic, WQS, QGC, and BKMR subgroup analyses were conducted using the fully adjusted models.

#### 2.5.6 Sensitivity analysis

As a sensitivity analysis, we excluded participants with severe liver disease (having alanine transaminase or aspartate transaminase levels exceeding 1,000 U/L) or kidney disease (having chronic kidney disease), defined based on previous studies [10,44,[47], [48], [49], [50]].

The threshold for statistical significance was set at a two-sided *P* value of less than 0.05. All analyses and visualizations were performed using R (Version 4.2.3; https://www.r-project.org).

## 3 Results

### 3.1 Population characteristics and concentrations of serum OCPs

Our study included a total of 6,529 participants, and the overall prevalence of hyperuricemia was 21.15%. Participants with hyperuricemia displayed certain demographic characteristics, such as being older, predominantly male, and having a higher representation of African American ethnicity. Additionally, these participants showed a higher prevalence of obesity and lower levels of renal function indicators (Table 1).

**Table 1:** Characteristics of the study population (N = 6,529) from NHANES, U.S., 2005–2016.

	Overall	Without hyperuricemia	With hyperuricemia	*P* value
**N**	6,529	5,148	1,381	
**Age, N (%)**				
20–50 years	3,342 (51.2)	2,807 (54.5)	535 (38.7)	**<0.001**
≥ 50 years	3,187 (48.8)	2,341 (45.5)	846 (61.3)	
**Sex, N (%)**				
Male	3,231 (49.5)	2,465 (47.9)	766 (55.5)	**<0.001**
Female	3,298 (50.5)	2,683 (52.1)	615 (44.5)	
**Race, N (%)**				
Hispanic-American	1,646 (25.2)	1,391 (27.0)	255 (18.5)	**<0.001**
Non-Hispanic White	2,962 (45.4)	2,319 (45.0)	643 (46.6)	
Non-Hispanic Black	1,317 (20.2)	966 (18.8)	351 (25.4)	
Other race	604 (9.3)	472 (9.2)	132 (9.6)	
**Education, N (%)**				
Less than high school	1,579 (24.2)	1,236 (24.0)	343 (24.8)	0.400
High school	1,475 (22.6)	1,150 (22.3)	325 (23.5)	
More than high school	3,475 (53.2)	2,762 (53.7)	713 (51.6)	
**BMI, N (%)**				
Normal weight	1,952 (29.0)	1,752 (33.1)	200 (14.0)	**<0.001**
Overweight	2,240 (33.3)	1,830 (34.5)	410 (28.7)	
Obese	2,537 (37.7)	1,718 (32.4)	819 (57.3)	
**Cotinine (ng/mL), median (IQR)**	0.04 [0.01, 8.34]	0.04 [0.01, 9.19]	0.05 [0.01, 4.36]	0.024
**Alcohol, N (%)**				
No	2,099 (27.5)	1,647 (27.3)	452 (28.2)	0.586
Yes	5,533 (72.5)	4,381 (72.7)	1,152 (71.8)	
**Poverty income ratio, N (%)**				
≤ 1.3	2,311 (30.3)	1,819 (30.2)	492 (30.7)	0.141
1.3–3.5	2,894 (37.9)	2,256 (37.4)	638 (39.8)	
> 3.5	2,427 (31.8)	1,953 (32.4)	474 (29.6)	
**Physical activity (MET time/week), N (%)**				
< 600	1,988 (30.4)	1,485 (28.8)	503 (36.4)	**<0.001**
600–1,199	500 (7.7)	395 (7.7)	105 (7.6)	
≥ 1,200	4,041 (61.9)	3,268 (63.5)	773 (56.0)	
**Total calorie intake (kcal per day), N (%)**				
Low	2,691 (41.2)	2,051 (39.8)	640 (46.3)	**<0.001**
Adequate	2,484 (38.0)	1,987 (38.6)	497 (36.0)	
High	1,354 (20.7)	1,110 (21.6)	244 (17.7)	
**Total frequency of seafood consumption, times**				
< 1	1,325 (20.3)	1,071 (20.8)	254 (18.5)	0.114
1–2	2,083 (31.9)	1,642 (31.9)	441 (32.0)	
2–3	705 (10.8)	546 (10.6)	159 (11.3)	
3–6	1,286 (19.7)	1,024 (19.9)	262 (19.0)	
≥ 6	1,130 (17.3)	865 (16.8)	265 (19.2)	
**Diabetes, N (%)**				
No	5,555 (85.1)	4,496 (87.3)	1,059 (76.7)	**<0.001**
Yes	974 (14.9)	652 (12.7)	322 (23.3)	
**Hypertension, N (%)**				
No	3,329 (51.0)	2,770 (53.8)	559 (40.5)	**<0.001**
Yes	3,200 (49.0)	2,378 (46.2)	822 (59.5)	
**Chronic kidney disease, N (%)**				
No	5,296 (81.1)	4,372 (84.9)	924 (66.9)	**<0.001**
Yes	1,233 (18.9)	776 (15.1)	457 (33.1)	
**Serum uric acid (mg/dL), mean (SD)**	5.50 (1.46)	4.96 (1.02)	**7.48 (1.06)**	**<0.001**

*P*-value for comparing the difference between participant with hyperuricemia and those without, given by t-tests for continuous variables and Chi-square tests for categorical variables. The numbers marked in bold indicate they are statistically significant.

Each of the five OCPs examined in this study exhibited a detection rate of at least 60% among the study population. The median and quartile concentrations of these chemicals are shown in Table S1. It's evident from the data that p,p′-DDE not only had the highest detection frequency but its concentrations also exceeded those of β-HCH, HCB, p,p′-DDT, and trans-nonachlor by over 20 times. The correlation among the concentrations of the five chemicals is statistically significant (*P* < 0.001). Notably, the most pronounced correlation was observed between p,p′-DDE and p,p′-DDT (*r*_*s*_ = 0.83), while the weakest correlation existed between p,p′-DDT, and trans-nonachlor (*r*_*s*_ = 0.37, Fig. S2).

### 3.2 Evaluating the association between single OCP exposure and hyperuricemia using weighted multiple logistic regression

**Fig. 1:**
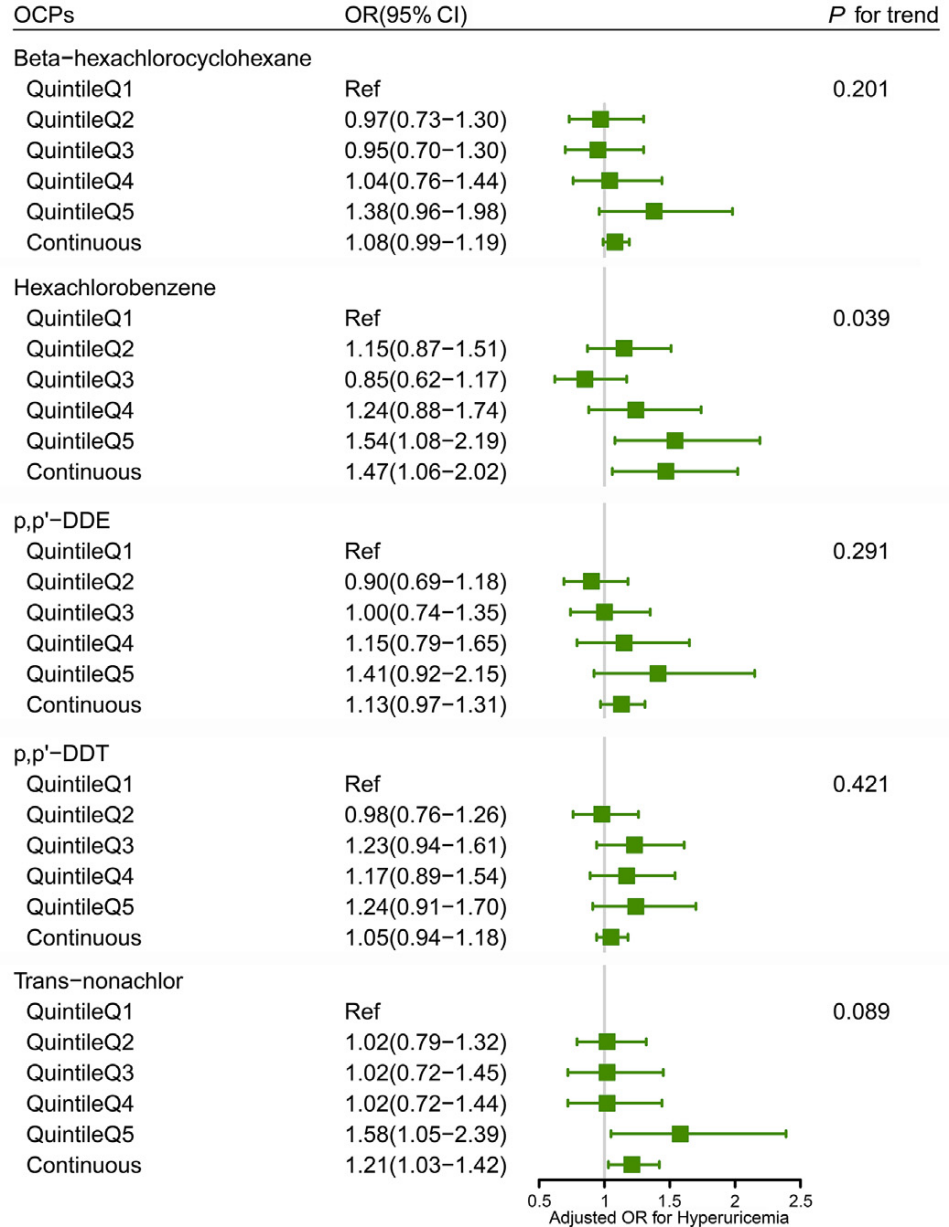
Association between five (organochlorine pesticide) exposures and hyperuricemia using weighted logistic regression. Adjusting age, sex, race, education level, BMI, PIR, serum cotinine concentration, alcohol consumption, physical activity, total frequency of seafood consumption, total calories taken, and medical history (diabetes, hypertension, CKD) (Model 3). BMI, body mass index; CI, confidence interval; CKD, chronic kidney disease; OR, odds ratio; PIR, poverty income ratio.

Significant dose–response effects were found in HCB (*P*-trend for HCB = 0.039) after adjusting for all covariates (Model 3). The ORs for hyperuricemia are statistically significant for the fifth quintile of exposure to HCB (OR: 1.54, 95% CI: 1.08–2.19) and trans-nonachlor (OR: 1.58, 95% CI: 1.05–2.39) compared to the lowest quintile. HCB and trans-nonachlor were included as continuous variables in the weighted logistic regression model, which also revealed a significant association with hyperuricemia (OR: 1.47, 95% CI: 1.06–2.02; OR: 1.21, 95% CI: 1.03–1.42, respectively), as shown in Figs. 1, S3 and S4. However, no significant association was observed between p,p′-DDT, p,p′-DDE, β-HCH, and hyperuricemia. Fig. S5 shows the sensitivity analysis results. In the fully adjusted model, HCB and trans-nonachlor still showed a positive association with hyperuricemia.

### 3.3 Evaluating the association between OCPs and hyperuricemia using WQS regression

The combined effect of the OCP mixture on hyperuricemia was initially modeled using WQS regression. Table 2 shows the results of three models adjusting for various covariates. In Model 1, without any variable adjustment, there is a significant positive relationship between the WQS index and hyperuricemia (OR: 1.41, 95% CI: 1.31–1.51). The positive association persisted even after adjusting for age, sex, BMI, race, PIR, and education level in Model 2 (OR: 1.28, 95% CI: 1.12–1.45). Upon further adjustments for serum cotinine concentration, alcohol consumption, physical activity, seafood consumption, total calorie intake, and medical history, the results remained significant in Model 3 (OR: 1.25, 95% CI: 1.09–1.44). These findings underscore a consistent positive relationship between the OCP mixture exposure and hyperuricemia.

**Table 2:** The association between OCP mixture and hyperuricemia using WQS and QGC (*N* = 6,529).

Method	Model	OR	95% CI of OR	*P* value
WQS	Model 1	1.41	(1.31, 1.51)	< 0.001
	Model 2	1.28	(1.12, 1.45)	0.002
	Model 3	1.25	(1.09, 1.44)	0.001
QGC	Model 1	1.37	(1.28, 1.46)	< 0.001
	Model 2	1.22	(1.08, 1.38)	0.002
	Model 3	1.20	(1.06, 1.37)	0.004

Model 1: no variables adjusted. Model 2: adjusted for sex, age, race, education level, BMI, and PIR. Model 3: adjusted for age, sex, race, education level, BMI, PIR, serum cotinine concentration, alcohol consumption, physical activity, total frequency of seafood consumption, total calorie intake, and medical history (diabetes, hypertension, chronic kidney disease). OR estimates represent the odds ratios of hyperuricemia when the QGC index was increased by one quartile. QGC, Quantile g-computation; WQS, weighted quantile sum.

**Fig. 2:**
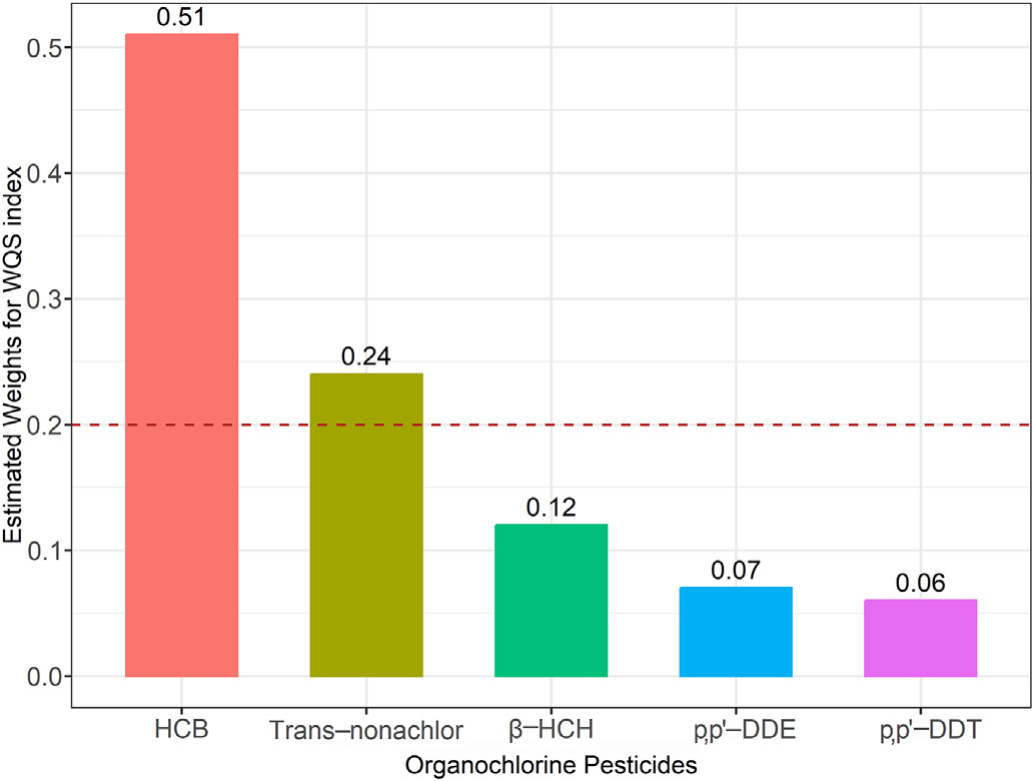
WQS model regression index weights for hyperuricemia. Model was adjusted for age, sex, race, education level, BMI, PIR, serum cotinine concentration, alcohol consumption, physical activity, total frequency of seafood consumption, total calories taken, and medical history (diabetes, hypertension, chronic kidney disease). The dashed line represents the prespecified cut-off established to identify the most important OCPs of the mixture and set equal to the inverse of the number of the mixture components (0.20). eGFR, estimated glomerular filtration rate; HCB, hexachlorobenzene; β-HCH, β-hexachlorocyclohexane.

The weights of the various chemicals in the WQS index in the fully adjusted model (Model 3) are displayed in Fig. 2, with HCB ranked first (0.51), followed by trans-nonachlor (0.24), then β-HCH (0.12), and p,p′-DDT exhibiting the lowest weight (0.06).

To explore the potential modifiers, stratified analyses were performed on age, sex, and PIR (Table S2). Specific subgroups exhibited a notable and favorable relationship between the WQS index and hyperuricemia, including females, individuals aged over 50 years, and those with a PIR below 3.5, as revealed by the study.

Table S4 shows the sensitivity analysis results. In the fully adjusted WQS model (Model 3), combined OCP exposure still showed a significant positive association with hyperuricemia (OR: 1.26, 95% CI: 1.07–1.48).

### 3.4 Evaluating the association between OCPs and hyperuricemia using the QGC model

The combined effect of the OCP mixture on hyperuricemia was initially modeled using the QGC model (Table 2). In Model 1, without any variable adjustment, there is a significant positive relationship between the mixed OCPs and hyperuricemia (OR: 1.37, 95% CI: 1.28–1.46). The positive association persisted after adjusting for age, sex, BMI, race, PIR, and education level in Model 2 (OR: 1.22, 95% CI: 1.08–1.38). Upon further adjustments for serum cotinine concentration, alcohol consumption, physical activity, seafood consumption, total calorie intake, and medical history, the results remained significant in Model 3 (OR: 1.20, 95% CI: 1.06–1.37). These findings underscore a consistent positive relationship between the OCP mixture exposure and hyperuricemia.

**Fig. 3:**
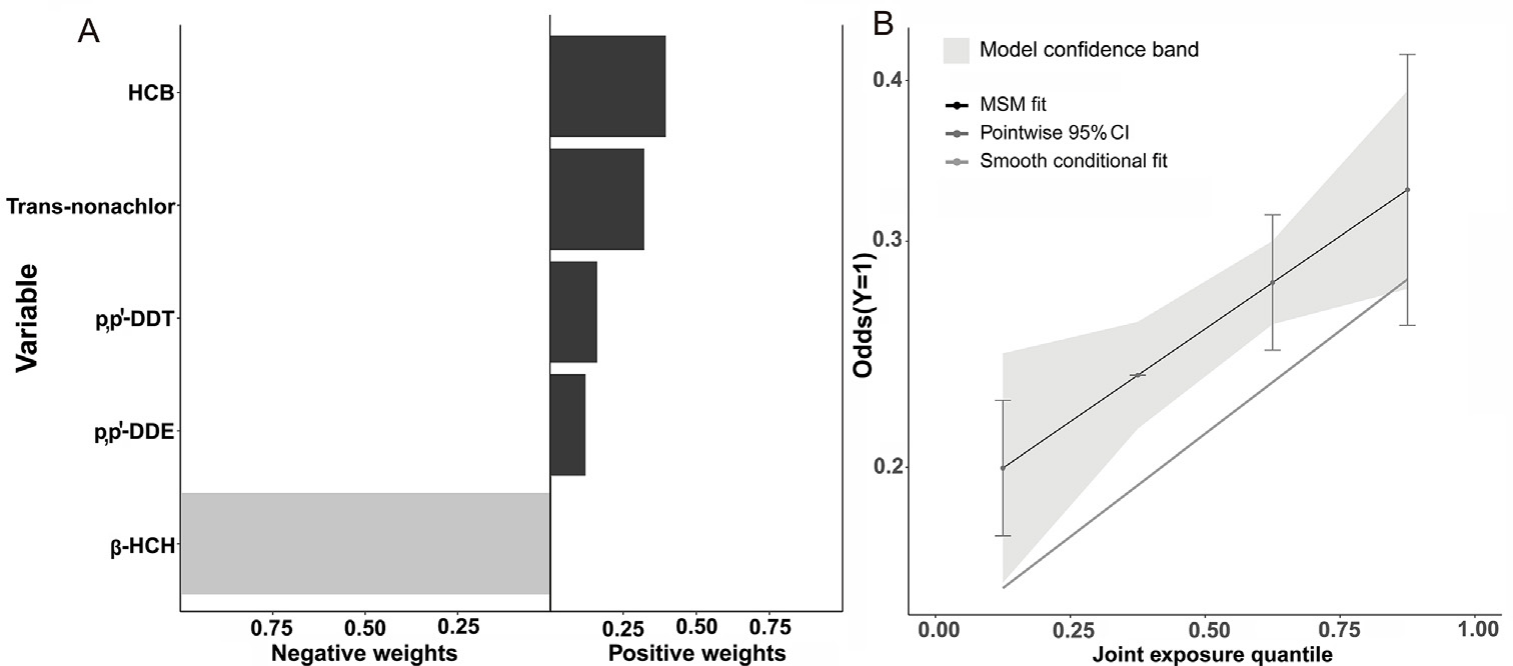
The proportion of positive and negative effects of OCPs on hyperuricemia and the combined effects of mixed exposure to OCPs in the QGC model. (A) Proportion effects of OCPs on hyperuricemia. (B) Combined effects for hyperuricemia.

The proportion of positive and negative effects of the various chemicals in the mixed OCPs in the fully adjusted model (Model 3) are displayed in Fig. 3A, with HCB ranked first, followed by trans-nonachlor, then p,p′-DDT, and β-HCH having negative weights. The QGC model also revealed a positive association between combined OCP exposure and hyperuricemia (Fig. 3B).

To explore the potential modifiers, stratified analyses were performed on age, sex, and PIR (Table S3). Specific subgroups exhibited a notable and favorable relationship between the OCP mixture exposure and hyperuricemia, including females, individuals aged over 50 years, and those with a PIR below 3.5, as revealed by the study.

Table S4 shows the sensitivity analysis results. In the fully adjusted QGC model (Model 3), combined OCP exposure still showed a significant positive association with hyperuricemia (OR: 1.18, 95% CI: 1.03–1.38).

### 3.5 Evaluating the association between OCPs and hyperuricemia using BKMR

Each OCP concentration underwent natural logarithm transformation and was analyzed as a continuous variable. The BKMR model was then used to validate the joint effect of OCP exposure on hyperuricemia. The posterior inclusion probability (PIP) was derived from the BKMR model for each chemical. Remarkably, HCB was the most important OCP component with the highest PIP of 0.938, while β-HCH exhibited the lowest PIP of 0.026 (Table S5).

**Fig. 4:**
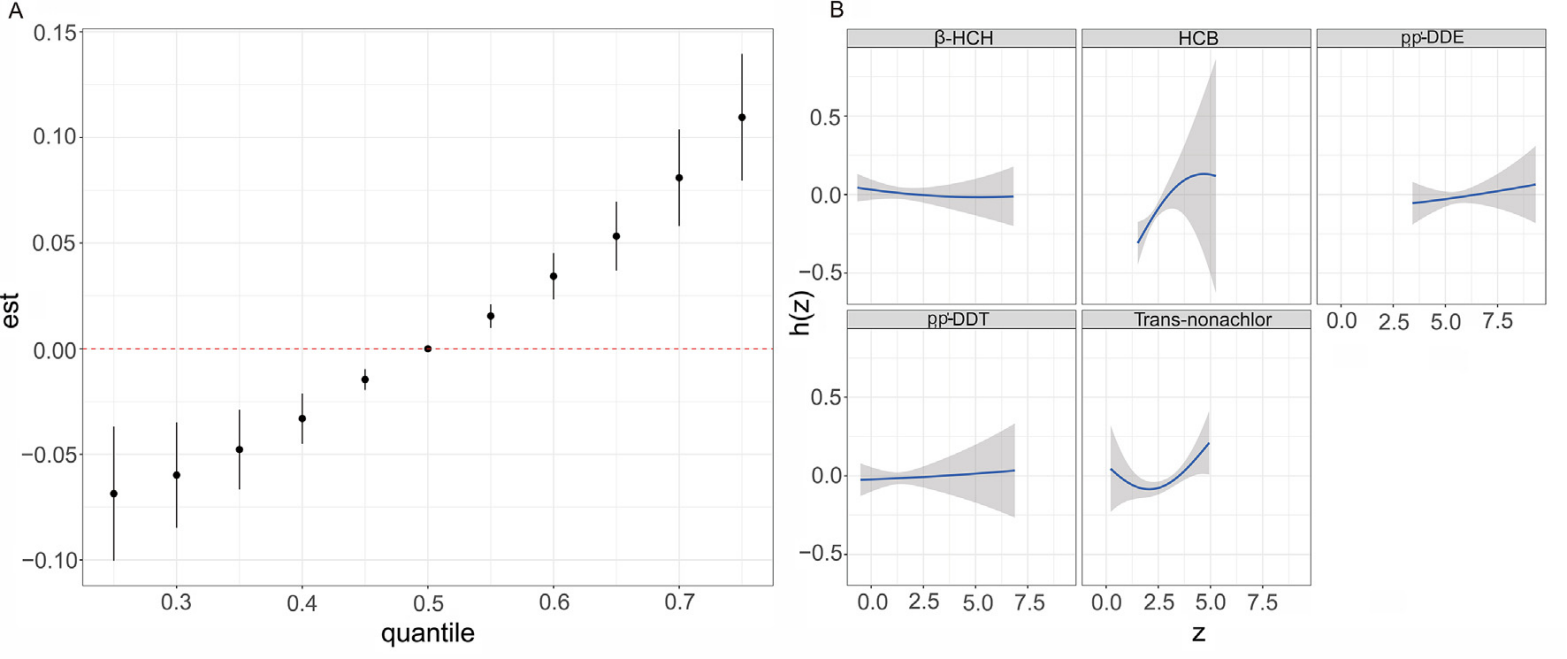
Association between OCP's exposure and hyperuricemia by hierarchical BKMR model. h(z) can be interpreted as the relationship between OCPs and hyperuricemia. The model was adjusted for age, sex, race, BMI, education level, PIR, alcohol consumption, serum cotinine concentration, physical activity, total frequency of seafood consumption, total calories taken, and medical history (diabetes, hypertension, CKD). (A) Joint effects (95% CI) of mixed OCP's exposure on hyperuricemia estimated by comparing all OCPs at particular percentiles (from 25th to 75th) with all the OCPs at their 50th percentiles. (B) Univariate exposure-response functions (95% CI) between each OCP concentration and hyperuricemia with fixing the concentrations of other OCPs at their 50th percentiles.

Comparing all chemicals at the 50th percentile, a significant positive linear correlation exists between the overall OCP mixture, and the prevalence of hyperuricemia was evident as the percentile concentration of OCP mixture exposure increased by 5 percentiles. For instance, OCPs set at their 60th, 70th, and 75th percentiles exhibited associations with β increases in the risk for hyperuricemia of 0.05 (0.04, 0.07), 0.08 (0.06, 0.10), and 0.11 (0.08, 0.14), respectively (Fig. 4A).

As for single pollutant concentration–response relationships, HCB showed a positive relationship with hyperuricemia when all other chemicals were at median levels (Fig. 4B). Except for a slight decline at low concentrations, trans-nonachlor shows an increased correlation with hyperuricemia as well. The remaining p,p′-DDT and p,p′-DDE also show a minor positive relationship with hyperuricemia, while β-HCH has a flat relationship.

We then estimated the variations in hyperuricemia risk associated with an increase in the interquartile range (IQR) of a single chemical pollutant while keeping others constant at the 25th, 50th, or 75th percentile levels (Fig. S6). We found that HCB and trans-nonachlor showed a positive and significant impact. The association between HCB and hyperuricemia seemed to be greater at higher percentiles of other chemicals. Specifically, when assigning values to the remaining pollutants based on the 25th, 50th, and 75th percentiles, a notable correlation existed between alterations in the IQR of HCB concentration and a rise in the prevalence of hyperuricemia. The corresponding values for this association were 0.07 (95% CI: 0.01–0.13), 0.08 (95% CI: 0.03–0.13), and 0.10 (95% CI: 0.04–0.15). Similar relationships were also observed for trans-nonachlor when other pollutants were set at the 50th and 75th percentiles.

To investigate the interactions among the five OCPs, we plotted their bivariate exposure-response functions (Fig. S7). The results suggested potential interactions between trans-nonachlor and the other four OCPs in their associations with hyperuricemia. Specifically, a stronger positive association between HCB, p,p′-DDE and hyperuricemia was observed when trans-nonachlor levels were higher (row 4, columns 1 and 2), while a more negative trend in the slope between p,p′-DDT, β-HCH, and hyperuricemia risk emerged when trans-nonachlor levels were higher (row 4, columns 3 and 5).

Additionally, we further performed hypothesis tests of potential interactions using the CVEK method. The ten pairwise generalized score tests (Table S6) indicated statistically significant interactions between trans-nonachlor and HCB, p,p′-DDE, β-HCH (with both *P* < 0.001). However, the interaction test between trans-nonachlor and p,p′-DDT did not reach statistical significance (*P* = 0.11).

Subgroup analyses revealed significant positive associations between exposure to mixed OCPs and hyperuricemia among females, individuals with a PIR of less than 3.50 and those aged 50 and older (Fig. S8). Sensitivity analysis results, as depicted in Fig. S9, were consistent with the overall findings in the study population.

## 4 Discussion

Despite the ban on the production of OCPs decades ago, our study indicates that most OCPs remain detectable in the general U.S. population. Many previous epidemiological and experimental studies have documented the adverse chronic health effects of OCPs on metabolic syndrome (MetS) and its components [51,52]. Surprisingly, despite numerous research demonstrating significant correlations between serum UA concentration and MetS (and its components) [[53], [54], [55]], there have been relatively few studies examining the association between OCPs and hyperuricemia [1,16,17,56]. Therefore, the potential impact of OCP exposure on the general population remains uncertain. In the current study, drawing from a large NHANES sample and employing multiple statistical models, we reported the detrimental impacts of OCPs on hyperuricemia.

From an analytical perspective, we first identified a positive relationship between HCB, trans-nonachlor, and hyperuricemia using weighted multiple logistic regression analyses. Subsequently, the WQS and QGC models highlighted a statistically significant correlation between the overall effect (denoted as OCP mixture exposure) and hyperuricemia, with HCB being the major contributor. Lastly, the BKMR analyses confirmed this notable correlation and the most important contributors.

Multiple logistic regression is a commonly used method due to its rapid modeling, straightforward outcomes, and ease of interpretation when exploring the health effects of environmental chemicals [27]. Several epidemiological studies using logistic regression have reported an association between OCPs and hyperuricemia, consistent with our findings [12,16,17]. However, logistic regression typically focuses on individual environmental chemicals or similar ones, neglecting mixture exposure to environmental chemicals, leading to potentially biased estimates [41]. In our analysis, for instance, we observed medium to strong correlations among the majority of OCP components, highlighting the importance of considering co-exposure to other pollutants to avoid confounding effects.

The WQS regression model is a widely used approach for analyzing mixed exposures because it more accurately replicates complex real-world exposures, such as the high correlation and interactions between environmental chemicals. Compared to the analysis of individual chemicals, the WQS regression model can identify important components more robustly under conditions where multicollinearity may exist among various OCP mixtures. In our analyses, the fully adjusted WQS models not only demonstrated a significant detrimental impact of OCP mixture exposure on hyperuricemia, but also determined the contributions of each OCP to hyperuricemia, addressing queries that could not be answered by multiple logistic regression [40]. However, the WQS regression model has limitations as well. On one hand, the model scores exposures using quantiles, which may result in the loss of some subtle exposure information [19]. On the other hand, the WQS model requires that all environmental chemicals included have the same impact direction [41]. QGC, as an extension of the traditional WQS method without assuming directional homogeneity, overcomes such limitations. It assesses the synergistic impacts of numerous compounds, combining the adaptability of g-computation with the simplicity of WQS regression. This makes QGC a robust framework for estimating unbiased mixture effects [20], thereby providing valuable insights into the joint effects of mixtures.

The BKMR model is a novel approach for examining mixed exposure. It evaluates the exposure-response function while considering the presence of other fixed chemicals at specific concentrations, as well as the whole-body impact of chemicals with varying orientations [57]. Through our analysis, a statistically significant association between the overall mixed exposure and hyperuricemia was identified. The positive relationships between HCB, trans-nonachlor, and hyperuricemia were consistent with the findings of the multiple logistic regression model. Contrarily, β-HCH showed a flat association with hyperuricemia, aligning with its statistically insignificant odds ratio in the logistic regression and its low weight in the QGC regression model. However, the kernel algorithm used by the BKMR model presents a limitation [27]. Estimating the co-exposure patterns of chemicals at levels other than the fixed level (the median level in this study) poses challenges when fitting the exposure-response function alongside other chemicals. To date, there has been no direct comparison among the WQS, QGC, and BKMR models. Evaluations of the merits and limitations of these four methodologies could help resolve ongoing disputes in environmental exposure research.

Our study employed weighted logistic regression, WQS, QGC, and BKMR methods, which have been widely used in previous similar research [27,30,43,58]. However, it is important to acknowledge that these methods for investigating mixed exposure effects have their own strengths and limitations, and there is no consensus on a singular best approach. Therefore, discrepancies in the results obtained from different analytical methods might be common. We believe it is essential to integrate findings from multiple methods to draw comprehensive conclusions. In our study, the consistent identification of HCB and trans-nonachlor as significant chemicals by WQS, QGC, and BKMR methods highlights their potential detrimental impacts. These components, standing out across different analytical approaches, warrant further research and attention in environmental management and policy-making.

Intriguingly, our analyses indicate potential interactions between trans-nonachlor and the other four OCPs. This finding is consistent with the complex nature of OCP interactions in biological systems. Previous studies suggest that the health impacts of OCPs can vary significantly depending on specific compounds and their interactions [59,60]. The potential antagonism or synergism observed between trans-nonachlor and other OCPs might stem from competitive interactions at the receptor level or differential metabolic pathways. These interactions could modulate the overall toxicological profile of these compounds, underscoring the importance of considering combined and interactive effects in assessing health risks associated with OCP exposures [1]. However, the mechanisms of these interactions should be verified by further in vivo or human studies [61].

According to the application of the aforementioned four statistical methods, we consistently found a significant association between OCP exposure and hyperuricemia. The potential mechanisms might be explained in the following aspects: (1) Many previous studies suggest that the primary cause of hyperuricemia is a decrease in the excretion of UA, which is the end product of purine metabolism and mainly filtered through the glomeruli in the kidneys and excreted in urine [62]. Exposure to OCPs might lead to damage to glomerular cells, decreasing the glomerular filtration rate and reducing UA clearance ability; (2) OCPs may influence the homeostasis of UA metabolism by interfering with antioxidant enzymes and subsequently generating reactive oxygen intermediates [1]. Exposure to OCPs may induce alternative glutathione detoxification pathways, which is linked to an increase in oxidative stress [63]; (3) Interactions between serum OCPs and the anion transport proteins involved in renal excretion can lead to chronic nephrotoxicity, interfering with UA excretion [64]; (4) The excretion of both UA and metabolic by-products of OCPs are both controlled by the renal transport excretion system, potentially leading to competition between OCPs and UA for kidney transport proteins [65]. Previous findings indicated that exposure to OCPs might disrupt the homeostasis of UA and kidney function, obstructing UA excretion, leading to its accumulation in the blood, and increasing the risk of hyperuricemia, gout, and other diseases.

Additionally, our subgroup analyses offered unique insights into OCP exposures in different populations. In the WQS, QGC, and BKMR models, we found significant associations between mixed OCP exposure and hyperuricemia in females and those with low PIR. Previous studies have indicated that in the female population, serum concentrations of most OCPs tend to be higher than in males, which may be attributed to differences in body fat percentage, dietary habits, metabolism, specific activities, and OCP contamination of gender dimensions [[66], [67], [68], [69], [70]]. Moreover, prior research has highlighted the significant burden of OCPs among populations in the U.S. with lower socio-economic status, these populations primarily engage in high-risk occupations such as agriculture, forestry, and fishing, increasing their risk of coming into contact with OCPs [[71], [72], [73], [74]].

Our study exhibits several notable strengths. Firstly, it represents the initial attempt to elucidate the association between exposure to mixed OCPs and hyperuricemia with a large population-based sample. The statistical power of our analyses has been enhanced due to the utilization of a substantial sample size over more than a decade and the adequate adjustment of variables, resulting in increased reliability of the findings. Secondly, the advantages of the four statistical methodologies complement one another. Specifically, we can explore a single relationship between an OCP and hyperuricemia using weighted multiple logistic regression. The WQS and QGC models provide a further assessment of the overall impact of OCPs on hyperuricemia. Additionally, the BKMR model confirms the relationship between total OCP exposure and the exposure-response function, as well as the exposure to each OCP. Our results, acquired using four different methodologies, appear more consistent and persuasive compared to previous studies. Thirdly, our subgroup analyses revealed that women, the elderly, and low-income populations were more sensitive to OCPs. Furthermore, further investigation through mechanistic studies is warranted to better understand the underlying pathways involved.

In comparison to our study, there are some previous similar ones. For instance, Zheng et al. [75] conducted a comprehensive study encompassing 4,973 adults and 1,381 adolescents, utilizing data from the NHANES spanning 2003 to 2018. Employing multivariate logistic regression, restricted cubic spline models, and WQS regressions, the study suggested a significant negative association between serum concentrations of perfluorooctanoate (PFDA) (OR: 0.65, 95% CI: 0.50–0.85) and total Per- and Polyfluoroalkyl Substances (PFAS) (OR: 0.92, 95% CI: 0.85–0.99) with the prevalence of MetS in adults. Parallel findings were observed in adolescents, revealing inverse correlations between the prevalence of MetS and levels of PFDA (OR: 0.55, 95% CI: 0.38–0.80), perfluorooctanoic acid (PFOA) (OR: 0.62, 95% CI: 0.39–1.00), perfluorooctane sulfonic acid (PFOS) (OR: 0.59, 95% CI: 0.36–0.96), and total PFAS (OR: 0.61, 95% CI: 0.37–0.99). On the other hand, Zha et al. [76] focused on urinary metal exposure and liver function, analyzing data from 8,158 NHANES participants between 2005 and 2018. Their analysis, employing methods including weighted multiple linear regression and BKMR, indicated a significant positive correlation of cadmium exposure with liver function, while cobalt exposure was inversely related to serum total bilirubin elevation. The main strength of our study is that we integrated findings from weighted logistic regression, WQS, QGC, and BKMR to make a more robust and in-depth exploration of the associations between OCP exposures and hyperuricemia. It is important to acknowledge that methods for studying mixed exposure effects in environmental epidemiology each have their strengths and limitations, and there is no consensus on a singular best approach. Therefore, we suggest using multiple analytical approaches to find the most detrimental pollutants and to avoid reporting false positive associations when assessing the synergistic effects of multiple pollutants.

There are also several limitations. First, a diet rich in purine would increase uric acid levels, potentially impacting hyperuricemia diagnosis. Because of the limited information on daily diet, we did not include dietary habits in our study. Second, although a wide range of confounders were adjusted in our study, we were unable to eliminate effects from other unreported confounders (e.g., occupation, medication history, and family history). Third, we utilized unweighted data when applying WQS, QGC, and BKMR models because these models do not support the integration of complex sample weights in their standard implementations [44,77,78]. Future methodological improvements integrating complex survey designs in these models are needed. Fourth, when concentrations fell below the minimum detectable level, a constant value was substituted. In addition, because hyperuricemia was diagnosed based on a single measurement and lacked disease history and medication information, the causal relationship between OCP exposure and hyperuricemia remained elusive, due to the inherent limitations of cross-sectional surveys. Large prospective cohort studies and experimental studies are further required to provide additional substantial evidence.

## 5 Conclusion

In this study, we employed multiple statistical methods, including weighted logistic, WQS, QGC, and BKMR models, to comprehensively investigate the association between mixed exposure to OCPs and hyperuricemia. Based on the results of these four models, we concluded that the body burden of five OCPs significantly increased the risk of hyperuricemia, with HCB and trans-nonachlor emerging as the most significant detrimental chemicals for hyperuricemia. Furthermore, females, the elderly, and low-income populations might be more vulnerable to OCP's exposure. Our research highlights the importance of ongoing monitoring of OCP's concentrations in the general population. We call for the adoption of multiple analytic approaches and a collaborative interpretation of their results to enhance the reliability of conclusions drawn from such investigations.

## CRediT authorship contribution statement

Y.W.: conceptualization, analyses, visualization, writing–original draft. Y.B.N.W: writing–review & editing, methodology. R.J.C.: data preparation, validation, supervision. Y.G., J.L.P.: interpretation of results, visualization. J.W.L.: validation, analyses, data curation. H.X.J.: data preparation, validation, analyses. Z.Y.W.: validation, supervision, writing–review & editing, funding acquisition, project administration. All authors have read and agreed to the published version of the manuscript.

## Declaration of competing interests

The authors have declared no conflicts of interest.
