# Supplementary material for: Association between exposure to a mixture of organochlorine pesticides and hyperuricemia in U.S. adults: A comparison of four statistical models
Source: Eco Environ Health. 2024 Mar 5;3(2):192–201. doi: 10.1016/j.eehl.2024.02.005 (PMC11031731; doi:10.1016/j.eehl.2024.02.005)
Supplement: Multimedia component 1 [file mmc1.docx]

**Supplementary materials**

**Table S1** Description of five OCP levels among participants (N = 6,529), NHANES, U.S., 2005–2016.

|  |  | **Hyperuricemia** |  |  |
| --- | --- | --- | --- | --- |
| **OCPs (ng/g lipid)** | **Overall** | **No** | **Yes** | ***P* value** |
|  | 6529 | 5148 | 1381 |  |
| β-HCH (median [IQR]) | 5.41 [2.12, 15.70] | 5.11 [1.92, 14.61] | 6.81 [2.64, 18.40] | **<0.001** |
| HCB (median [IQR]) | 9.60 [8.00, 12.10] | 9.49 [7.85, 11.70] | 10.20 [8.29, 12.85] | **<0.001** |
| p,p'-DDE (median [IQR]) | 320.50 [135.00, 737.70] | 300.80 [132.80, 705.00] | 392.75 [165.00, 816.93] | **<0.001** |
| p,p'-DDT (median [IQR]) | 3.73 [2.33, 7.1] | 3.60 [2.28, 7.04] | 4.09 [2.60, 7.33] | **<0.001** |
| trans-nonachlor (median [IQR]) | 17.28 [8.15, 31.30] | 16.15 [7.76, 28.43] | 22.68 [10.89, 42.93] | **<0.001** |

Abbreviation: OCP: organochlorine pesticide; β-HCH: β-Hexachlorocyclohexane; HCB: Hexachlorobenzene; IQR: Inter-Quartile Range.

**Table S2** Association between OCP mixture and hyperuricemia using WQS in different subgroups, NHANES, U.S., 2005–2016.

| **Subgroup** | **OR (95%CI)** | ***P* Value** |
| --- | --- | --- |
| **Sex** |  |  |
| male | 1.09 (0.89, 1.34) | 0.418 |
| female | 1.38 (1.10, 1.76) | **0.004** |
|  |  |  |
| **Age** |  |  |
| under 50 years | 0.96 (0.82, 1.12) | 0.587 |
| over 50 years | 1.24(1.07, 1.43) | **0.007** |
|  |  |  |
| **PIR** |  |  |
| ≤1.3 | 1.37(1.17, 1.60) | **<0.001** |
| 1.3-3.5 | 1.26 (1.08, 1.47) | **0.003** |
| >3.5 | 1.09 (0.92, 1.30) | 0.308 |

WQS models were adjusted for age, sex, race, education level, BMI, PIR, serum cotinine concentration, alcohol consumption, physical activity, total frequency of seafood consumption, total calories take and medical history (diabetes, hypertension, chronic kidney disease). OR estimates represent the odds ratios of hyperuricemia when the WQS index was increased by one quartile.

Abbreviation: WQS: weighted quantile sum; OR: odds ratio; CI: confidence interval; BMI: body mass index; PIR: poverty income ratio; eGFR: estimated glomerular filtration rate.

**Table S3** Association between OCP mixture and hyperuricemia using QGC in different subgroups, NHANES, U.S., 2005–2016.

| **Subgroup** | **OR (95%CI)** | ***P* Value** |
| --- | --- | --- |
| **Sex** |  |  |
| male | 0.99 (0.83, 1.18) | 0.932 |
| female | 1.30 (1.06, 1.60) | **0.013** |
|  |  |  |
| **Age** |  |  |
| under 50 years | 1.12 (0.95, 1.31) | 0.161 |
| over 50 years | 1.16 (1.01, 1.34) | **0.030** |
|  |  |  |
| **PIR** |  |  |
| ≤1.3 | 1.32 (1.03, 1.68) | **0.026** |
| 1.3-3.5 | 1.25 (1.03, 1.54) | **0.024** |
| >3.5 | 1.10 (0.89, 1.35) | 0.385 |

QGC models were adjusted for age, sex, race, education level, BMI, PIR, serum cotinine concentration, alcohol consumption, physical activity, total frequency of seafood consumption, total calories take and medical history (diabetes, hypertension, chronic kidney disease). OR estimates represent the odds ratios of hyperuricemia when the QGC index was increased by one quartile.

Abbreviation: QGC: Quantile g-computation; OR: odds ratio; CI: confidence interval; BMI: body mass index; PIR: poverty income ratio; eGFR: estimated glomerular filtration rate.

**Table S4** The separate sensitive analysis of association between OCP mixture and hyperuricemia using WQS and QGC.

| **Method** | **Model** | **OR** | **95% CI of OR** | ***P* value** |
| --- | --- | --- | --- | --- |
| **WQS** | Model 1 | 1.35 | (1.26, 1.44) | < **0.001** |
|  | Model 2 | 1.28 | (1.12, 1.46) | < **0.001** |
|  | Model 3 | 1.26 | (1.07, 1.48) | **0.005** |
| **QGC** | Model 1 | 1.19 | (1.10, 1.29) | **< 0.001** |
|  | Model 2 | 1.19 | (1.03, 1.37) | **0.013** |
|  | Model 3 | 1.18 | (1.03, 1.38) | **0.019** |

Model 1: no variables adjusted. Model 2: adjusted for sex, age, race, education level, BMI, PIR. Model 3: adjusted for age, sex, race, education level, BMI, PIR, serum cotinine concentration, alcohol consumption, physical activity, total frequency of seafood consumption, total calories take and medical history (diabetes, hypertension, chronic kidney disease). OR estimates represent the odds ratios of hyperuricemia when the QGC index was increased by one quartile.

Abbreviation: QGC: Quantile g-computation; OR: odds ratio; CI: confidence interval; BMI: body mass index; PIR: poverty income ratio; eGFR: estimated glomerular filtration rate.

**Table S5** Posterior inclusion probabilities (PIPs) into hyperuricemia models, using BKMR model (*N* = 6,529).

| **Rank** | **OCPs** | **PIP** |
| --- | --- | --- |
| 1 | HCB | 0.938 |
| 2 | trans-nonachlor | 0.714 |
| 3 | p,p'-DDE | 0.167 |
| 4 | p,p'-DDT | 0.079 |
| 5 | β-HCH | 0.026 |

Abbreviation: β-HCH: β-Hexachlorocyclohexane; HCB: Hexachlorobenzene; BKMR: Bayesian kernel machine regression.

**Table S6** The ten pairwise generalized score tests of potential interactions using the CVEK method

| **Pairwise generalized score tests** | ***P* value** |
| --- | --- |
|  |  |
| HCB and trans-nonachlor | **< 0.001** |
| HCB and p,p'-DDE | 0.98 |
| HCB and p,p'-DDT | 0.87 |
| HCB and β-HCH | 0.79 |
| trans-nonachlor and p,p'-DDE | **< 0.001** |
| trans-nonachlor and p,p'-DDT | 0.11 |
| trans-nonachlor and β-HCH | **< 0.001** |
| p,p'-DDE and p,p'-DDT | 0.85 |
| p,p'-DDE and β-HCH | 0.93 |
| p,p'-DDT and β-HCH | 0.66 |

Abbreviation: β-HCH: β-Hexachlorocyclohexane; HCB: Hexachlorobenzene; CVEK: Cross-validated Ensemble of Kernels.

**Figure S1** Flowchart of participants selection (N = 6529), NHANES, U.S., 2005–2016.

**Figure S2** Pairwise Spearman correlations among urinary concentrations of five organochlorine pesticides in the population (N = 6,529), NHANES, U.S., 2005-2016. All the correlations were statistically significant (*P* < 0.001).

**Figure S3** Association between five (organochlorine pesticide) exposures and hyperuricemia using weighted logistic regression without adjusting any confounding factors (Model 1).

Abbreviation: OR: odds ratio; CI: confidence interval.

**Figure S4** Association between five (organochlorine pesticide) exposures and hyperuricemia using weighted logistic regression, adjusting age, sex, race, education level, PIR, and BMI (Model 2).

Abbreviation: OR: odds ratio; CI: confidence interval; PIR: poverty income ratio; BMI: body mass index.

**Figure S5** The separate sensitive analysis: association between five (organochlorine pesticide) exposures and hyperuricemia using weighted logistic regression, adjusting age, sex, race, education level, BMI, PIR, serum cotinine concentration, alcohol consumption, physical activity, total frequency of seafood consumption, total calories take and medical history (diabetes, hypertension, CKD).

Abbreviation: OR: odds ratio; CI: confidence interval; BMI: body mass index; PIR: poverty income ratio; CKD: chronic kidney disease.

**Figure S6** Single-pollutant association with hyperuricemia. The plot shows the change in risks of hyperuricemia with a 95% confidence interval (CI) in a single OCP, when all other OCPs were fixed at the 25th, 50th, or 75th percentile.

**Figure S7** The trend of exposure-response in three quantiles, the function of a single exposure 1 where the other exposure 2 was fixed at 0.1, 0.5, 0.9 quantiles, and other exposures were fixed at their 50th percentiles. The results were assessed by the BKMR model, adjusted for age, sex, race, education level, BMI, PIR, serum cotinine concentration, alcohol consumption, physical activity, total frequency of seafood consumption, total calories take and medical history (diabetes, hypertension, CKD).

**Figure S8** Overall effects of environmental exposures on hyperuricemia in different subgroups. Fig. 7A shows the sex groups (A1: Male, A2: Female). Fig. 7B shows the PIR groups (B1: <1.30, B2: 1.3-3.5, B3: >3.5). Fig. 7C shows the age groups (C1: 20-50 years, C2: ≥ 50 years).

**Figure S9** Association between OCPs exposure and hyperuricemia by hierarchical BKMR model in the separate sensitive analysis. h(z) can be interpreted as the relationship between OCPs and hyperuricemia. The model was adjusted for age, sex, race, BMI, education level, PIR, alcohol consumption, serum cotinine concentration, physical activity, total frequency of seafood consumption, total calories take and medical history (diabetes, hypertension, CKD). (A) Joint effects (95% CI) of mixed OCPs exposure on hyperuricemia estimated by comparing all OCPs at particular percentiles (from 25th to 75th) with all the OCPs at their 50th percentiles. (B) Univariate exposure-response functions (95% CI) between each OCP concentration and hyperuricemia with fixing the concentrations of other OCPs at their 50th percentiles.
